# The Molecular Basis of the Interaction of Cyclophilin A with α‐Synuclein

**DOI:** 10.1002/anie.201914878

**Published:** 2020-01-29

**Authors:** Filippo Favretto, Jeremy D. Baker, Timo Strohäker, Loren B. Andreas, Laura J. Blair, Stefan Becker, Markus Zweckstetter

**Affiliations:** ^1^ Translational Structural Biology in Dementia German Center for Neurodegenerative Diseases (DZNE) Von-Siebold-Str. 3a 37075 Göttingen Germany; ^2^ Department of Molecular Medicine Morsani College of Medicine USF Health Byrd Alzheimer's Institute University of South Florida Tampa FL 33613 USA; ^3^ Department for NMR-based Structural Biology Max Planck Institute for Biophysical Chemistry Am Fassberg 11 37077 Göttingen Germany

**Keywords:** cyclophilin, Parkinson's disease, proline isomerization, protein structure, α-synuclein

## Abstract

Peptidylprolyl isomerases (PPIases) catalyze cis/trans isomerization of prolines. The PPIase CypA colocalizes with the Parkinson's disease (PD)‐associated protein α‐synuclein in cells and interacts with α‐synuclein oligomers. Herein, we describe atomic insights into the molecular details of the α‐synuclein/CypA interaction. NMR spectroscopy shows that CypA catalyzes isomerization of proline 128 in the C‐terminal domain of α‐synuclein. Strikingly, we reveal a second CypA‐binding site formed by the hydrophobic sequence ^47^GVVHGVATVA^56^, termed PreNAC. The 1.38 Å crystal structure of the CypA/PreNAC complex displays a contact between alanine 53 of α‐synuclein and glutamine 111 in the catalytic pocket of CypA. Mutation of alanine 53 to glutamate, as found in patients with early‐onset PD, weakens the interaction of α‐synuclein with CypA. Our study provides high‐resolution insights into the structure of the PD‐associated protein α‐synuclein in complex with the most abundant cellular cyclophilin.

## Graphical abstract

**The high‐resolution structure** of the PreNAC region of α‐synuclein in complex with the peptidylprolyl isomerase cyclophilin A provides a novel entry point for the modulation of α‐synuclein‐related neurotoxicity. The A53E mutation of α‐synuclein, which causes early‐onset Parkinson's disease, weakens the interaction of α‐synuclein with cyclophilin A.

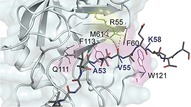

Intrinsically disordered proteins (IDPs) are important for a wide range of biological processes and human disorders.^1^ A characteristic property of IDPs is their high content of prolines.^2^ In the cell, *cis*/*trans* isomerization of prolines is catalyzed by peptidylprolyl isomerases (PPIases).^3^ One family of PPIases is the cyclophilins with cyclophilin A (CypA) making up approximately 0.1–0.6 % of the total cytosolic proteins.^4^ CypA plays an important role in several human diseases including neurodegeneration.^5^ In agreement with an important role of CypA for neurodegeneration, the activity of PPIases has been associated with pathogenic aggregation of IDPs.^6^

α‐Synuclein (α‐syn) is a paradigmatic IDP associated with Parkinson's disease (PD).^7^ Most of the familial mutations that cause early‐onset PD are located in the segment ^47^GVVHGVATVA^56^, termed PreNAC.7a The PreNAC and the hydrophobic sequence ^68^GAVVTGVTAVA^78^ in the central part contribute to α‐syn‐binding to membranes and form the core of amyloid fibrils.^8^ The C‐terminal domain of α‐syn is involved in protein–protein interactions^9^ and contains five proline residues that are important for α‐syn aggregation.^10^ Insoluble inclusions in the brain of patients with α‐synucleinopathies are positive for both α‐syn and PPIases.^11^ CypA influences the aggregation of α‐syn in vitro and colocalizes with α‐syn in cells.^12^ Furthermore, co‐immunoprecipitation showed that CypA interacts with α‐syn oligomers in cells.^13^ Little is known, however, about the molecular details of the α‐syn/CypA interaction.

α‐Syn contains the five proline residues P108, P117, P120, P128, and P138 in the acidic C‐terminal domain (Figure 1 a). *cis*‐Pro populations of the five X–Pro bonds are less than 5 % (Figure 1 b).2b, 14 NMR spectra are thus dominated by the *trans* conformers of α‐syn (Figure 1 c; blue). The addition of increasing CypA concentrations caused progressive broadening of selected α‐syn cross‐peaks with some additional changes in peak position (Figure 1 c; orange). Residue‐specific analysis identified two main CypA‐binding regions in α‐syn (Figure 1 d): E46–Q62 in the central part of α‐syn and V118–E131 in the C‐terminal proline‐rich region (Figure 1 d). Smaller NMR signal perturbations were also observed in the approximate vicinity of residues V26 and V77 (Figure 1 d), suggesting additional, weaker CypA‐binding to these regions.

**Figure 1:**
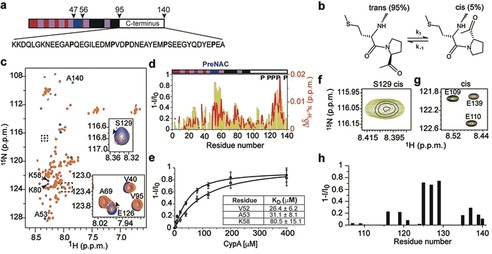
CypA binding and catalysis of *cis*/*trans* isomerization in α‐syn. a) Domain organization of α‐syn: N‐terminal amphipathic region (red), NAC (non‐amyloid‐β component, black, and acidic C‐terminal domain (white). The PreNAC region (residues 47–56) is displayed in blue. b) Schematic representation of *cis*/*trans* isomerization of M127–P128 in α‐syn. c) ^1^H‐^15^N HSQC of α‐syn alone (blue) and with 5‐fold excess of CypA (orange). d) Intensity changes (yellow bars) and chemical‐shift changes Δδ_1H‐15_N (red line) in α‐syn upon addition of a 5‐fold excess of CypA. I_0_ and I are the intensities of ^1^H‐^15^N HSQC cross‐peaks in the absence and presence of CypA, respectively. The locations of the five proline residues (P) of α‐syn are marked. e) Intensity changes of V52 (black square) and K58 of α‐syn (open squares), which have predominantly slow exchange behavior, in the presence of increasing CypA concentrations. The error bars are derived from signal‐to‐noise ratios in NMR spectra. The lines represent best fits to the experimental data assuming a reversible 1‐to‐1 binding model. f,g) Cross‐peaks of *cis* conformers of S129 (f) and three C‐terminal glutamic acids (g) in the absence (green) and presence of CypA (black). The α‐syn:CypA molar ratio was 100:1. h) Site‐specific intensity decrease of the *cis* cross‐peaks of α‐syn upon addition of CypA.

In the C‐terminus, changes in signal intensity as well as signal position were observed, suggesting that the interaction of this region with CypA is intermediate on the NMR‐chemical‐shift time scale (Figure 1 d). In contrast, the region E46–Q62 experienced predominantly signal broadening (Figure 1 d). On the basis of their slow exchange behavior, dissociation constants (*K*_D_) from 26–80 μm were derived for V52, A53, and K58 (Figure 1 e). Combined analysis resulted in a *K*_D_ of 41±6 μm. The CypA‐binding region in the central part overlaps with the PreNAC region of α‐syn (G47–A56; Figure 1 a). Notably, the PreNAC region does not contain proline residues.

At high protein concentrations, the low‐populated *cis* conformers of α‐syn become observable in ^1^H‐^15^N correlation spectra (Figure 1 f,g).^14^ The addition of substoichiometric concentrations of CypA (CypA:α‐syn molar ratio of 1:100) strongly attenuated the intensity of the cross‐peaks of the *cis* conformers of Y125, M127, and S129, that is, residues in the direct vicinity of P128 (Figure 1 h). In contrast, little broadening was observed close to the other four proline residues (P108, P117, P120, P138). The data show that CypA predominantly catalyzes the *cis*/*trans* isomerization of P128.

To identify the α‐syn‐binding site in CypA, we performed NMR titrations with ^15^N‐labeled CypA (Figure 2 a). With increasing α‐syn concentrations, the signals of several CypA residues shifted and broadened. CypA signal perturbation reports on the binding process of α‐syn and is caused by binding‐induced changes in the chemical environment and local dynamics of CypA as well as by the exchange between the α‐syn‐bound and free form of CypA residues. CypA signal changes saturated at approximately 10‐fold excess of α‐syn over CypA. Residue‐specific analysis located the α‐syn‐induced changes to residues within or in proximity to the catalytic site (Figure 2 b and Supporting Information, Figure S1 a). Furthermore, peptides corresponding to the PreNAC and C‐terminal proline‐rich region induced NMR signal perturbations in the shallow binding interface of CypA (Supporting Information, Figure S1 b–g). Both the C‐terminal proline‐rich region and the central PreNAC thus bind to the catalytic binding pocket of CypA.

**Figure 2:**
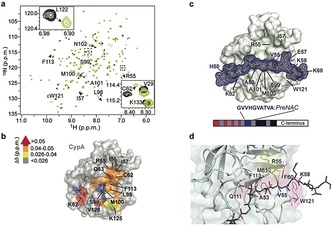
Atomic resolution structure of α‐syn PreNAC bound to CypA. a) ^1^H‐^15^N HSQC of CypA alone (black) and in presence of a 5‐fold excess of α‐syn (green). Selected CypA cross‐peaks are labeled. b) Mapping of residue‐specific chemical‐shift changes observed in (a) onto the structure of CypA (from gray/Δδ${}_{{}^{1}H\text{-}{}^{15}N}$ <0.026 ppm to red/Δδ${}_{{}^{1}H\text{-}{}^{15}N}$ >0.05 ppm). Residues with strong signal attenuation are shown in blue. c) Crystal structure of the CypA/PreNAC complex (2 *m* *F*_o_−*D* *F*_c_ electron density map of PreNAC contoured at 2σ level, depicted in blue). d) Expansion of the binding site of CypA in complex with the PreNAC region of α‐syn. CypA interface residues are labeled in black, α‐syn residues in blue.

To gain atomic insight into the interaction of α‐syn with CypA, we crystallized the complex of CypA with the α‐syn^PreNAC^ peptide (Supporting Information, Tables S1 and S2). Within this structure, residues V48–K60 of α‐syn^PreNAC^ are tightly bound to the shallow substrate‐binding pocket of CypA (Figure 2 c). The complex structure revealed a contact between the side chain of A53 of α‐syn^PreNAC^ and the carbon tail of Q111, as well as hydrogen bonding between the carbonyl group of V55 of α‐syn and the R55 guanidino side chain group of CypA (Figure 2 d). Furthermore, the hydrophobic CypA residues F60, M61, and F113 surround the side chain of V55 (Figure 2 c,d). Further stabilization of the complex is provided by an intermolecular contact between the side‐chain carbons of K58 and the CypA‐residue W121 (Figure 2 d).

To test the contribution of the catalytically important R55 side chain, we titrated the R55A‐mutant of CypA to α‐syn (Supporting Information, Figure S2).^15^ The addition of the R55A‐mutant of CypA induced almost no signal broadening in the C‐terminal proline‐rich region of α‐syn (Supporting Information, Figure S2 b). Binding to the PreNAC of α‐syn still occurred, but was attenuated when compared to unmodified CypA (Supporting Information, Figure S2 b).

The PreNAC region contains most of the mutations in α‐syn that have so far been associated with familial PD (Figure 3 a).7a We prepared the α‐syn mutants H50Q, G51D, and A53E and probed their interaction with CypA. The CypA‐interaction profiles of both H50Q α‐syn and G51D α‐syn closely superimpose with that of the wild‐type protein (Supporting Information, Figure S3 a,b). An unperturbed interaction profile is consistent with the structure of the PreNAC/CypA complex (Figure 3 b): the two α‐syn residues H50 and G51 are positioned at the end of the binding pocket of CypA, where mutations are expected to only weakly affect complex formation.

**Figure 3:**
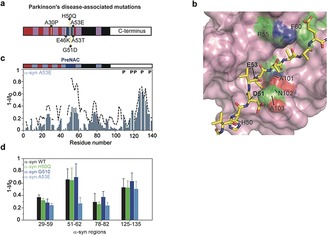
PD‐associated mutation in α‐syn modulates CypA binding. a) Location of PD‐associated mutations in α‐syn. Most mutations are located in the PreNAC region. b) Modelling of the α‐syn mutations A53E and G51D in the context of the 3D structure of the CypA/α‐syn^PreNAC^ complex. CypA and PreNAC are shown in purple and yellow, respectively. The introduced side chains E53 and D51 are labeled in bold. c) Comparison of residue‐specific intensity changes in wild‐type (dashed line) and A53E mutant (blue bars) α‐syn upon addition of a 5‐fold excess of CypA. d) Region‐specific CypA‐induced signal broadening in α‐syn (black) and disease‐associated variants. Error bars were derived on the basis of NMR signal‐to‐noise ratios.

In the structure of the PreNAC/CypA complex, the side chain of A53 is intimately involved in the binding interface (Figure 2 d). The PD‐associated mutation A53E introduces a negative charge into this binding interface and sterically clashes with CypA residues (Figure 3 b). Indeed, the A53E mutation weakens the interaction of the PreNAC region with CypA and shifts the binding process from the slow to the intermediate exchange regime (Figure 3 c,d). In contrast, the interaction of CypA with the proline‐rich region of α‐syn is unaffected (Figure 3 c,d).

Aggregation of α‐syn plays a central role in the development of PD. Within the 140‐residue sequence of α‐syn, the PreNAC region is a pathogenic hot spot, because it harbors most of the disease‐associated mutations (Figure 3).7a, 16 We found that CypA binds to the PreNAC (Figure 1). The high‐resolution structure of the α‐syn^PreNAC^ peptide in complex with CypA showed that the hydrophobic valine at position 55 of α‐syn is intimately involved in the binding to CypA. Notably, the PreNAC sequence does not contain a proline residue, consistent with previous studies demonstrating that the presence of proline is not a strict requirement to bind to the shallow architecture of the catalytic pocket of PPIase domains.3a In the context of PD and other synucleinopathies, our finding that the A53E mutation, which causes early‐onset PD, strongly attenuates the interaction of α‐syn with CypA suggests that changes in the interaction of α‐syn with PPIases and other molecular chaperones as a result of patient‐associated mutations or post‐translational modifications play an important role in α‐syn‐mediated neurotoxcity. The high‐resolution structure of PreNAC in complex with CypA (Figure 2) can therefore provide a novel entry point for the modulation of α‐syn‐induced neurotoxicity in neurodegenerative disorders.

## Conflict of interest

The authors declare no conflict of interest.

## Supporting information

As a service to our authors and readers, this journal provides supporting information supplied by the authors. Such materials are peer reviewed and may be re‐organized for online delivery, but are not copy‐edited or typeset. Technical support issues arising from supporting information (other than missing files) should be addressed to the authors.

Supplementary
