# Supplementary material for: The Molecular Basis of the Interaction of Cyclophilin A with α‐Synuclein
Source: Angew Chem Int Ed Engl. 2020 Jan 29;59(14):5643–6. doi: 10.1002/anie.201914878 (PMC7085457; doi:10.1002/anie.201914878)
Supplement: Supplementary file 1 — Supplementary [file ANIE-59-5643-s001.pdf]

Angewandte Chemie
Eine Zeitschrift der Gesellschaft Deutscher Chemiker

Supporting Information

# The Molecular Basis of the Interaction of Cyclophilin A with α-Synuclein

*Filippo Favretto, Jeremy D. Baker, Timo Strohäker, Loren B. Andreas, Laura J. Blair, Stefan Becker,* and Markus Zweckstetter**

anie_201914878_sm_miscellaneous_information.pdf

## Supplementary Figures

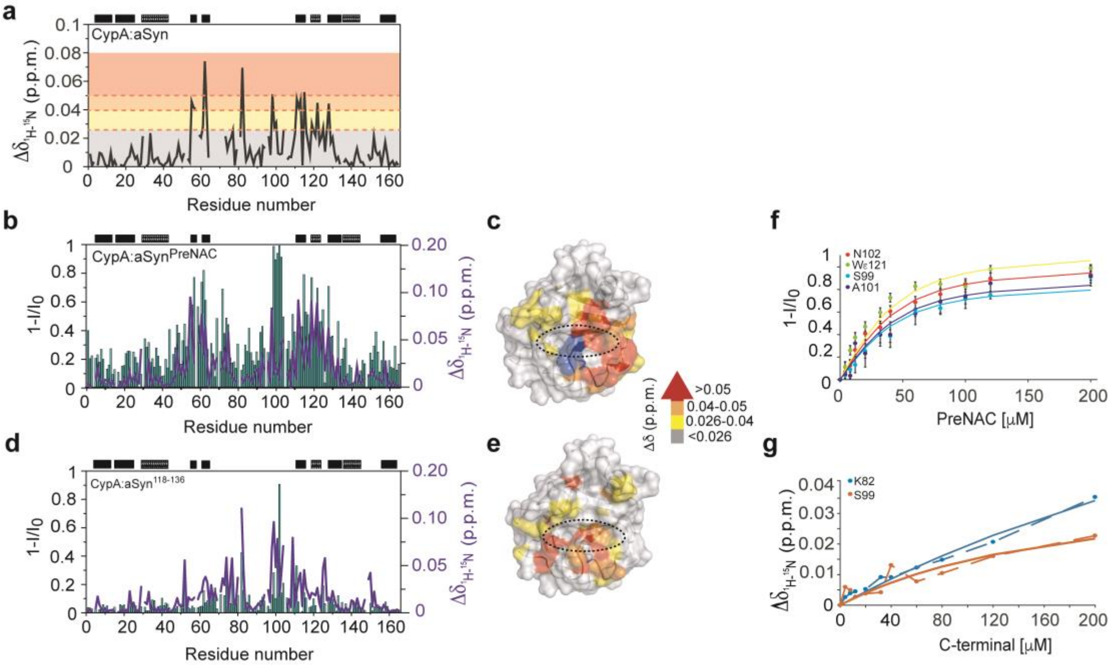


**Figure S1.** Residue-specific chemical shift perturbation in CypA upon binding to aSyn. **a)** Residue-specific chemical shift changes in CypA upon addition of a 5-fold excess of aSyn. Regions undergoing chemical shift changes are mapped in Fig. 2b onto the 3D structure of CypA from grey ($\Delta\delta_{1H-15N} < 0.026$ ppm) to red ($\Delta\delta_{1H-15N} > 0.05$ ppm). CypA secondary structure elements are shown on top. **b-e)** Intensity changes (green bars) and chemical shift changes $\Delta\delta_{1H-15N}$ (purple line) in $^{1}$H-$^{15}$N HSQC spectra of CypA in presence of 8-fold molar excess of the peptides aSyn$_{PreNAC}$ (b,c) and aSyn$_{118-136}$ (d,e). CypA residues, which were broadened beyond detection in presence of aSyn$_{PreNAC}$, are shown in blue in (c). **f)** Changes in signal intensities of selected CypA residues at increasing concentrations of the peptide aSyn$_{PreNAC}$. **g)** Changes in chemical shifts of selected CypA residues at increasing concentrations of the peptide aSyn$_{118-136}$.

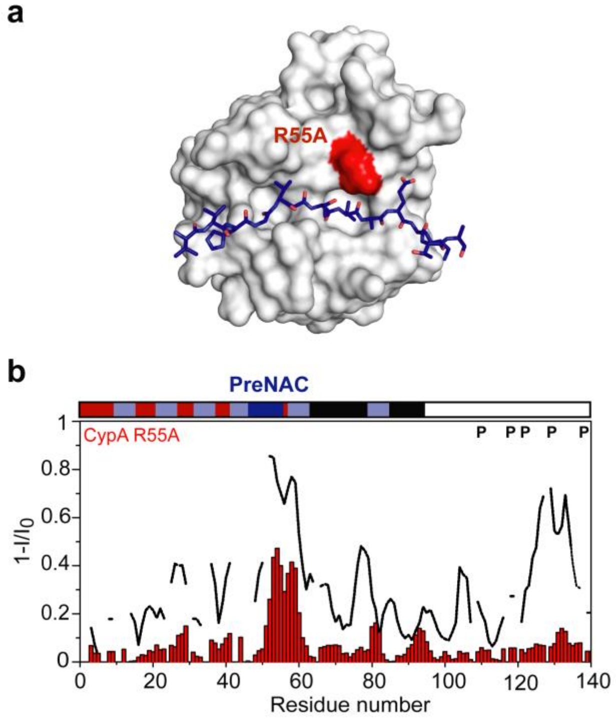


**Figure S2.** Mutation of the catalytic CypA residue R55 attenuates binding to aSyn. **a)** Location of the CypA mutation R55A in the 3D structure of the CypA/aSyn$_{\mathrm{PreNAC}}$ complex. **b)** Residue-specific intensity changes in aSyn upon addition of a 5-fold excess of CypA$_{\mathrm{R55A}}$ (red bars). $I_0$ and I are the intensities of $^{1}$H-$^{15}$N HSQC cross-peaks in the absence and presence of CypA$_{\mathrm{R55A}}$, respectively. For comparison, the black line displays the intensity broadening profile induced by wild-type CypA at the same molar ratio.

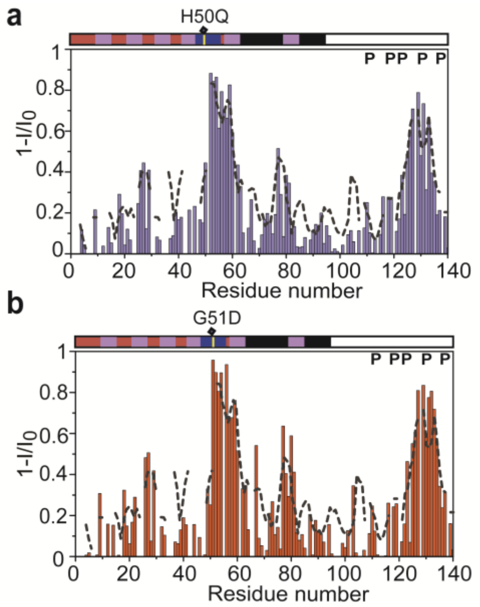


**Figure S3.** Binding of CypA to aSyn containing PD-associated mutations H50Q (a) and G51D (b). Residue-specific intensity changes upon addition of a 5-fold excess of CypA. The CypA-induced intensity broadening profile of wild-type aSyn is shown as dashed line.

**Table S1. X-ray data collection statistics.**

| **Data statistics** | **PreNac/CypA-complex** |
|---|---|
| Wavelength | 1.0 Å |
| Beamline | SLS-X10SA |
| Detector | PILATUS 6M |
| Space group | $P4_32_12$ |
| *a* | 61.034 Å |
| *b* | 61.034 Å |
| *c* | 129.152 Å |
| Resolution[a] | 1.38 Å (1.40-1.38 Å) |
| Reflections measured | 1,231,308 |
| Unique reflections | 51,144 |
| Redundancy | 24.01 (12.56) |
| Completeness(%) | 99.7 (92.6) |
| Mean $I/\sigma(I)$ | 27.39 (1.16) |
| $R_{rim}$ (%)[b] | 2.92 (68.9) |

[a] Values in parentheses are outer-resolution shell.

[b] $R_{rim} = \sum_{hkl} \left[ N/(N-1) \right]^{1/2} \sum_i \left| I_i(hkl) - \langle I(hkl) \rangle \right| / \sum_{hkl} \sum_i I_i(hkl)$, where N is the redundancy and $I_i(hkl)$ is the ith observation of reflection hkl and ⟨I(hkl)⟩ is the weighted average intensity for all observations i of reflection hkl.

**Table S2. X-ray structure refinement statistics CypA/αSyn$_{PreNAC}$-complex.**

| | |
|---|---|
| *R*-factor[a] | 16.3% |
| $R_{free}$[b] | 17.8% |
| Solvent | 60.6% |
| Mean B-value (Å$^2$) | |
| chain A | 24.26 |
| chain B | 32.01 |
| waters | 37.5 |
| No. of protein residues | 177 |
| No. of water residues | 248 |
| **Root mean square deviations from ideal geometry** | |
| Bond lengths | 0.019 Å |
| Bond angles | 2.18° |
| **Ramachandran plot (%)** | |
| Favoured | 95.1 |
| Allowed | 4.9 |
| Outliers | 0 |

[a] $R = \sum_{hkl} \left| |F_{obs}| - |F_{calc}| \right| / \sum_{hkl} |F_{obs}|$ , where $F_{obs}$ and $F_{calc}$ are the observed and calculated structure factors, respectively.

[b] $R_{free}$ was determined using 5% of the data [1].

## Supplementary Experimental Section

Protein expression and purification of aSyn proteins was performed as described previously.[17] aSyn peptides were synthesized by solid-phase peptide synthesis. The gene of human CypA was cloned into a modified pET28a vector (Addgene) and site-directed mutagenesis was carried out using a QuikChange kit (Qiagen). CypA and its R55A variant were recombinantly expressed in *Escherichia coli* BL21(DE3) cells (Novagen) as described in [18]. Proteins were dialyzed against the NMR buffer containing 100 mM NaCl, 50 mM HEPES, 0.02 % $NaN_3$, pH 7.4.

NMR experiments were recorded on 600, 700, 800, 900, 950 MHz Bruker NMR spectrometers. For backbone resonance assignment of $^{15}N$/$^{13}C$-labeled CypA, 3D HNCA, HNCACB, HNCO, HNCACO and $^{15}N$-edited NOESY-HSQC (NOESY

mixing time: 120 ms) experiments were recorded. NMR-based interaction studies were acquired at 15 °C and the combined $^{1}H/^{15}N$ chemical shift perturbation $(((\delta_H)^2 + (\delta_N/5)^2)/2)^{1/2}$ was calculated. The intensities were fitted assuming a simple two state exchange model and the $K_d$ was calculated according to:

$$\left(1 - \frac{I}{I_0}\right) = I_{max}\left[\frac{(P_0 + x + K_d) - \sqrt{(P_0 + x + K_d)^2 - 4P_0x}}{2P_0}\right]$$

where $I$ is the intensity value along the titration, $I_0$ is the intensity value of the free state, $P_0$ is the total amount of protein, $K_d$ is the dissociation constant and $x$ the concentration of CypA in µM along the titration. Errors were estimated by evaluating the standard deviation of the intensity according to:

$$\sigma_I = \left(\frac{I}{I_0}\right)\sqrt{(\frac{\sigma I}{I})^2 + (\frac{\sigma I_0}{I_0})^2}$$

where $\sigma I$ and $\sigma I_0$ are the standard deviations of the noise in the spectra. To identify the *cis*-proline conformers, aSyn (600 µM) was resuspended in 20 mM phosphate buffer (0.02% $NaN_3$, 2 mM DTT, pH 6.0).

For crystallization, the aSyn(E46-Q62) peptide was added in 4-fold molar excess to CypA. Total protein concentration was adjusted in NMR buffer to 20 mg/ml. Crystals were obtained at 20 °C by sitting drop vapor diffusion using 1.93 M tri-ammonium citrate, pH 7.0, as precipitant. For data collection crystals were soaked for 1 minute in 2.5 M tri-ammonium citrate, pH 7.0, as cryoprotectant. Data collection was performed at SLS Villigen, Switzerland (beamline PXII, Pilatus 6M detector [19]). Data were processed with XDS.[20] Space group determination and statistical analysis was performed with XPREP (Bruker AXS, Madison, Wisconsin, USA). The structure was solved by molecular replacement with PHASER [21] using the crystal structure of CypA (PDB code: 5KUL [22]) as search model. Refinement was performed with Refmac[23] alternating with manual model building in Coot.[24]
